# Reliability of endoscopic dye transit test for prediction of functional success after diode laser and external dacryocystorhinostomy

**DOI:** 10.5935/0004-2749.20200007

**Published:** 2020

**Authors:** Eduardo Damous Feijó, Juliana Alves Caixeta, Adriana Ribeiro de Almeida, Roberto Murillo Limongi, Suzana Matayoshi

**Affiliations:** 1 Department of Oculoplastic Surgery, Hospital Oftalmológico de Anápolis, Anápolis, GO, Brazil; 2 Department of Otorhinolaringology, Hospital Oftalmológico de Anápolis, Anápolis, GO, Brazil; 3 Hospital Oftalmológico de Anápolis, Anápolis, GO, Brazil; 4 Department of Oculoplastic Surgery, Universidade Federal de Goiás, Goiânia, GO, Brazil; 5 Department of Oculoplastic Surgery, Universidade de São Paulo, São Paulo, SP, Brazil

**Keywords:** Lacrimal apparatus diseases, Dacryocystorhinostomy, Endoscopy, Nasolacrimal duct, Laser therapy/methods, Lasers, semiconductor, Predictive value of tests, Doenças do aparelho lacrimal, Dacriocistorinostomia, Endoscopia, Ducto nasolacrimal, Terapia a laser/métodos, Lasers semicondutores, Valor preditivo dos testes

## Abstract

**Purpose:**

To determine the reliability of the endoscopic dye transit test for the prediction of functional success after dacryocystorhinostomy.

**Methods:**

A cross-sectional study was conducted with 50 patients who underwent external dacryocystorhinostomy Group or transcanalicular dacryocystorhinostomy Group and had anatomically patent ducts during irrigation, with a minimum 6-month follow-up. The external dacryocystorhinostomy, defined as the time from instillation of the dye into the conjunctival sac until its flow from the rhinostomy site, was performed in all patients. Positive predictive value of the endoscopic dye transit test to assess functional success was analyzed. The cutoff point was determined using a receiver operating characteristic curve.

**Results:**

Of the 50 patients, 44 (88%) exhibited subjective improvement or complete resolution of epiphora (functional success). The best cutoff point for the endoscopic dye transit test was 60 s. Of 39 patients with endoscopic dye transit test ≤60 s, 38 (97.4%) exhibited functional success, demonstrating a 97.4% positive predictive value.

**Conclusion:**

The endoscopic dye transit test ≤60 s is a reliable tool to predict functional success and good prognosis after external or laser transcanalicular dacryocystorhinostomy.

## Abstract (pt)

**Objetivo:**

Determinar a confiabilidade do teste endoscópico do corante na predição do sucesso funcional após dacriocistorrinostomia.

**Métodos:**

Estudo transversal com 50 pacientes submetidos ao grupo de dacriocistorrinostomia externa ou grupo dacriocistorrinostomia transcanalicular e que possuíam dutos anatomicamente patentes pela irrigação, com seguimento mínimo de 6 meses. A dacriocistorrinostomia externa, definida como o tempo desde a instilação do corante no saco conjuntival até o fluxo do local da rinostomia, foi realizada em todos os pacientes. O valor preditivo positivo do teste endoscópico do corante para avaliar o sucesso funcional foi analisado. O ponto de corte foi determinado usando uma curva característica de operação do receptor.

**Resultados:**

Dos 50 pacientes, 44 (88%) apresentaram melhora subjetiva ou resolução completa da epífora (sucesso funcional). O melhor ponto de corte para o teste endoscópico do corante foi de 60 s. Dos 39 pacientes com teste endoscópico do corante ≤60 s, 38 (97,4%) apresentaram sucesso funcional, demonstrando um valor preditivo positivo de 97,4%.

**Conclusão:**

O teste en doscópico do corante ≤60 s é uma ferramenta confiável para predizer o sucesso funcional e o bom prognóstico após dacriocistorrinostomia transcanalicular externa ou a laser.

## INTRODUCTION

Success after lacrimal surgery is poorly defined. The most practical measure of success is control of symptoms; however, this can be discordant with anatomic outcome^(1)^. There is a discrepancy between anatomic and functional success rates in dacryocystorhinostomy (DCR). Even when some tear ducts are patent to irrigation (anatomic success), the procedure does not necessarily improve tearing symptoms (functional failure)^(2^-^7)^. Approximately 10% of patients experience persistent epiphora following anatomically successful DCR.

Qualitative evaluation, including lacrimal syringing, fluorescein dye disappearance testing, air bubble testing, and functional endoscopic dye testing, may overestimate the functional success rates of the procedure. Few reports exist on quantitative assessment of lacrimal drainage. Slow postoperative tear transit can cause epiphora^(8)^, despite anatomic and objective functional postoperative success^(9)^.

External DCR (EDCR) remains the gold standard surgical technique for nasolacrimal duct obstruction (NLDO)^(10^-^13)^, with a 90% success rate^(2^-^4)^. It is possible to perform laser transcanalicular DCR (TDCR), but the surgical success rate of this type of DCR is lower than that of EDCR and endonasal DCR^(5^-^8)^. However, the absence of incisions in the orbicularis muscle and lacrimal sac could prevent lacrimal pump damage, resulting in better tear drainage in patients who have undergone TDCR.

In this context, we determined the reliability of endoscopic dye transit test (EDTT) for the prediction of functional success after EDCR or TDCR.

## METHODS

This cross-sectional study was performed on 50 p atients who underwent EDCR (25 patients) and TDCR (25 patients). All patients had patent ducts on lacrimal irrigation postoperatively, confirmed by positive syringing with no reflux from the opposite canaliculus. The minimum postoperative follow-up was 8 months^(14)^. All patients underwent the Jones 1 and syringing tests at all postoperative evaluations.

Inclusion criteria were EDCR or TDCR for the treatment of primary nasolacrimal duct obstruction (PANDO) and anatomic patency through irrigation postoperatively. Exclusion criteria were age <21 years, canalicular obstruction, common canalicular stenosis, trichiasis, ectropion, entropion, hypometric blink, lagophthalmos, secondary lacrimal obstruction, history of facial trauma, facial palsy, accentuated nasal septum deviation, middle turbinate hypertrophy nasal synechiae, and/or nasal polyps. The protocol was approved by the medical research ethics committee of the University of São Paulo (USP) and followed the guidelines established by the Declaration of Helsinki. All patients signed an informed consent form.

EDTT was defined as the time from instillation of 1 drop 2% fluorescein (Allergan, Dublin, Ireland) into the conjunctiva to its free flow from the ostium site. EDTT was assessed through nasofibroscopy using a 0º rigid endoscope (Storz, Tuttlingen, Germany)^(15^,^16)^. EDTT was measured in seconds using a stopwatch^(17)^. Functional success was defined as the resolution or improvement of epiphora (Munk score, 0 or 1).

Functional success (Munk score, 0-1) was assessed using a positive predictive test of EDTT. The cutoff point was 60 s, determined using a receiver-operating curve (the maximum sensibility and specificity point). The sample size was calculated from the target difference of 20% in EDTT between the 2 groups and pooled standard difference (SD) from a previous study (FTT, 45 s; SD, 10 s). A power of 80% and confidence level of 95% (95% CI) yielded a sample size of >19 in each arm

*P*<0.05 was considered statistically significant. All statistical evaluations were performed using SPSS software (SPSS, Inc., Chicago, IL, USA).

## RESULTS

The 50 patients included in the study were divided into 2 groups: the EDCR group (25 patients [20 women, 80%]; mean age, 58 years; range, 38-84 years; SD, 13.64) and the TDCR group (25 patients [19 women, 78%]; mean age, 56 years; range, 29-92 years; SD, 17.99; p=0.902). All patients had ducts that were anatomically patent to syringing with no reflux. Mean follow-up was 13 and 11 months in the EDCR and TDCR groups, respectively (p=0.332).

Functional success was 88% in the 2 groups. A total of 22 patients in each group (44 of 50, 88%) exhibited improvement or complete resolution of epiphora. Three patients in each group (12%) reported that postoperative epiphora was the same or had improved slightly (Munk score, 2). The discrepancy between anatomic (100%) and subjective functional success (88%) in our study was 12%.

Of 25 patients in the EDCR group, 17 had EDTT ≤60 s, and all of them had functional success (Munk score, 0 or 1). The positive predictive value (PPV) of the test to predict functional success in this group was 100% (p=0.042; Table 1).

**Table 1:** EDTT and functional success in the EDCR group

QFEDT/outcome	Success		Failure		Total
	N	%	N	%	
<60 sec	17	77.3	0	0	17
>60 sec	5	22.7	3	100	8
Total	22	100	3	100	25

*P*=0.042; PPV, 100%; Negative Predictive Value (NPV), 37.5%; Sensibility, 77.3%; Specificity, 100 %; relative risk (RR), 1.6 (95% CI, 1.01-2.737).

A total of 22 patients in the TDCR group had EDTT ≤60 s, and 21 demonstrated functional success (Munk score, 0 or 1). The PPV of the test to predict functional success in this group was 95.4% (*p*=0.029; Table 2).

**Table 2:** EDTT and functional success in the TDCR group

QFEDT/outcome	Success		Failure		Total
	N	%	N	%	
<60 sec	21	95.4	1	33.4	22
>60 sec	1	4.6	2	66.6	3
Total	22	100	3	100	25

*P*=0.021 ; PPV, 95.4%; NPV, 66.7%; Sensibility, 95.4 %; Specificity, 66.7 %; RR, 2.86 (95% CI, 1.570-14.093).

Considering all patients with functional success (44 of 50, 88%) and cases with EDTT ≤1 minute, 39 patients had an EDTT ≤1 minute and 38 of them had functional success. Of 6 patients with functional failure, 5 had EDTT >1 minute (p=0.003). EDTT ≤1 minute had a PPV of 97.4% (Table 3).

**Table 3:** EDTT and overall functional success

QFEDT/Outcome	Success		Failure		Total
	*N*	%	*N*	%	
<60 sec	38	86.3	1	16.7	39
>60 sec	6	13.7	5	83.3	11
Total	44	100	6	100	50

*P*=0.021 ; PPV, 97.4%; NPV, 45.4%; Sensibility, 86.3 %; Specificity, 83.3 %; RR, 1.78 (95% CI, 1.013-4.121).

There were no differences between the two groups with respect to EDTT.

## DISCUSSION

The PPV of EDTT ≤60 s to predict functional success was 97.4%. If we consider that the Jones 1 test (dye observed into the nose) was positive in 100% of all postoperative cases and the subjective functional success rate was 88% (12% misdiagnosis), and our test was better than the Jones 1 test.

Success after lacrimal surgery is poorly defined. The most practical measure of success is control of symptoms, although this can be discordant with anatomic outcome^(9)^. There is a discrepancy between anatomic and functional success rates in DCR. Anatomic success is determined primarily by patency to irrigation and a positive Jones I test, both qualitative tests. These assessments can overestimate success rates because the simple detection of dye from the nasal ostium is not a guarantee of functional success. Slow lacrimal drainage can cause epiphora^(14)^. In this study, 5 of 6 patients (83%) had EDTT >60 s, a finding that confirmed this hypo thesis. Almost all patients with EDTT ≤60 s exhibited functional success (97.4%). This result suggests that this test is a reliable tool to assess functional success after EDCR and TDCR because of its excellent PPV.^(16)^.

Lacrimal syringing and probing are invasive methods that do not easily diagnose functional failure, although they have been used widely to evaluate NLDO^(18)^. The fluorescein dye disappearance test (FDDT) is noninvasive, but it depends on subjective interpretation. Kashkouli et al.^(19)^ demonstrated that the FDDT had a PPV of 25.8%, although it had a sensitivity of 100%. Another quantitative lacrimal test is the measurement of tear meniscus height. The difficulty of this test is that it requires an anterior segment photograph and measurements of the number of pixels (using Photoshop), rendering the test impractical. Our test requires only fluorescein, a rigid nasal endoscope, and a stopwatch.

Approximately 10%-15% of patients continue to experience persistent epiphora following an anatomically successful DCR. This paradox has been explained by Rose^(9)^ who suggested that some patients have high resistance to drainage as a result of low hydraulic conductance through lacrimal pathways, resulting in watering despite anatomic patency. These cases are called “functional failures”^(13)^. The limitation of this study is the small number of patients in each arm and presence of older patients who cannot have good tear production. Patients can have no tearing because there is no production and not because the drainage is insufficient.

We conclude that EDTT is a reliable tool to predict functional success, and can be performed routinely to evaluate success after TDCR and EDCR.

- Dacryocystorhinostomy*/methods*
- Female
- Follow-Up Studies
- Humans
- Lasers, Semiconductor/therapeutic use*
- Male
- Middle Aged
- Nasolacrimal Duct/surgery*
- Retrospective Studies

## References

[1] Rose GE. (2004). The lacrimal paradox: toward a greater understanding of success
in lacrimal surgery. Ophthalmic Plast Reconstr Surg.

[2] Kaynak P, Ozturker C, Yazgan S, Karabulut GO, Akar S, Demirok A (2014). Transcanalicular diode laser assisted dacryocystorhinostomy in
primary acquired nasolacrimal duct obstruction: 2-year follow
up. Ophthalmic Plast Reconstr Surg.

[3] Savino G, Battendieri R, Traina S, Corbo G, D’Amico G, Gari M (2014). External vs. *endonasal* dacryocystorhinostomy:
has the current view changed?. Acta Otorhinolaryngol Ital.

[4] Tarbet KJ, Custer PL. (1995). External dacryocystorhinostomy. Surgical success, patient
satisfaction, and economic cost. Ophthalmology.

[5] Yoon SW, Yoon YS, Lee SH. (2006). Clinical results of endoscopic dacryocystorhinostomy using a
microdebrider. Korean J Ophthalmol.

[6] Akay F, Ilhan A, Yolcu U, Gundogan FC, Yildirim Y, Toyran S. (2015). Diode laser-assisted transcanalicular dacryocystorhinostomy: the
effect of age on the results. Arq Bras Oftalmol.

[7] Alanon Fernandez FJ, Alanon Fernandez MA, Martinez Fern andez A, Cardenas Lara M. (2004). [Transcanalicular dacryocystorhinostomy technique using diode
laser]. Arch Soc Esp Oftalmol.

[8] Dogan R, Meric A, Ozsutcu M, Yenigun A. (2013). Diode laser-assisted endoscopic dacryocystorhinostomy: a
comparison of three different combinations of adjunctive
procedures. Eur Arch Otorhinolaryngol.

[9] Maeso Riera J, Sellares Fabres MT. (2007). [Trans-canalicular diode laser dacryocystorhinostomy: technical
variations and results]. Acta Otorrinolaringol Esp.

[10] Alnawaiseh M, Mihailovic N, Wieneke AC, Prokosch V, Rosentreter A, Merte RL (2016). Long-Term outcomes of external dacryocystorhinostomy in the age
of transcanalicular microendoscopic techniques. J Ophthalmol.

[11] Kaynak P, Ozturker C, Yazgan S, Karabulut GO, Akar S, Demirok A (2014). Transcanalicular diode laser assisted dacryocystorhinostomy in
primary acquired nasolacrimal duct obstruction: 2-year follow
up. Ophthalmic Plast Reconstr Surg.

[12] Plaza G, Betere F, Nogueira A. (2007). Transcanalicular dacryocystorhinostomy with diode laser:
long-term results. Ophthalmic Plast Rec onstr Surg.

[13] Yildirim Y, Kar T, Topal T, Cesmeci E, Kaya A, Colakoglu K (2016). Comparison of transcanalicular multidiode laser
dacryocystorhinostomy with and without silicon tube
intubation. J Ophthalmol.

[14] Shams PN, Chen PG, Wormald PJ, Sloan B, Wilcsek G, McNab A (2014). Management of functional epiphora in patients with an
anatomically patent dacryocystorhinostomy. JAMA Ophthalmol.

[15] Delaney YM, Khooshabeh R. (2002). Fluorescein transit test time and symptomatic outcomes after
external dacryocystorhinostomy. Ophthalmic Plast Reconstr Surg.

[16] Ali MJ, Psaltis AJ, Wormald PJ. (2014). Dacryocystorhinostomy ostium: parameters to evaluate and DCR
ostium scoring. Clin Ophthalmol.

[17] Munk PL, Lin DT, Morris DC. (1990). Epiphora: treatment by means of dacryocystoplasty with balloon
dilation of the nasolacrimal drainage apparatus. Radiology.

[18] Roh JH, Chi MJ. (2010). Efficacy of dye disappearance test and tear meniscus height in
diagnosis and postoperative assessment of nasolacrimal duct
obstruction. Acta Ophthalmol.

[19] Kashkouli MB, Mirzajani H, Jamshidian-Tehrani M, Shahrzad S, Sanjari MS. (2015). Fluorescein dye disappearance test: a reliable test in assessment
of success after dacryocystorhinostomy procedure. Ophthalmic Plast Reconstr Surg.

